# Exposure to Nickel, Chromium, or Cadmium Causes Distinct Changes in the Gene Expression Patterns of a Rat Liver Derived Cell Line

**DOI:** 10.1371/journal.pone.0027730

**Published:** 2011-11-16

**Authors:** Matthew G. Permenter, John A. Lewis, David A. Jackson

**Affiliations:** 1 Excet, Inc., Fort Detrick, Maryland, United States of America; 2 U.S. Army Center for Environmental Health Research, Fort Detrick, Maryland, United States of America; Texas A&M University, United States of America

## Abstract

Many heavy metals, including nickel (Ni), cadmium (Cd), and chromium (Cr) are toxic industrial chemicals with an exposure risk in both occupational and environmental settings that may cause harmful outcomes. While these substances are known to produce adverse health effects leading to disease or health problems, the detailed mechanisms remain unclear. To elucidate the processes involved in the toxicity of nickel, cadmium, and chromium at the molecular level and to perform a comparative analysis, H4-II-E-C3 rat liver-derived cell lines were treated with soluble salts of each metal using concentrations derived from viability assays, and gene expression patterns were determined with DNA microarrays. We identified both common and unique biological responses to exposure to the three metals. Nickel, cadmium, chromium all induced oxidative stress with both similar and unique genes and pathways responding to this stress. Although all three metals are known to be genotoxic, evidence for DNA damage in our study only exists in response to chromium. Nickel induced a hypoxic response as well as inducing genes involved in chromatin structure, perhaps by replacing iron in key proteins. Cadmium distinctly perturbed genes related to endoplasmic reticulum stress and invoked the unfolded protein response leading to apoptosis. With these studies, we have completed the first gene expression comparative analysis of nickel, cadmium, and chromium in H4-II-E-C3 cells.

## Introduction

Many heavy metals, including nickel, chromium, and cadmium, are widely distributed, posing occupational and environmental exposure risks which may result in adverse health effects. Exposure to these metals can occur through contact with contaminated soil, air, water, and food, or by absorption through the skin as a result of manufacturing, pharmaceutical, or industrial processes or environmental contamination. Nickel is used extensively in many industrial and consumer products such as stainless steel, magnets, coins, and alloys; evidenced by the fact that 882 of the 1,662 current sites on the National Priorities List targeted for federal clean-up activities contain nickel [1]. Chromium is extensively used for stainless steel production, chrome plating, and pigments and is responsible for 500,000 industrial exposures in the United States [2], [3]. Exposure to cadmium can occur as a result of mining, metal processing, welding, burning fuels, the production and use of phosphate fertilizers, and leaching of metal waste, yet tobacco smoke and food are still the main sources of intake [4].

While many of the adverse health effects of nickel, cadmium, and chromium are similar, the exact mechanisms, modes of action, and biochemical pathways affected by each metal differ. For example, all three metals induce oxidative stress, but nickel and chromium undergo Fenton type reactions forming reactive oxygen species while cadmium is thought to cause oxidative stress through the inhibition of antioxidant enzymes [5], [6]. Similarly, all three metals have been shown to be genotoxic, but chromium is the only one of the three metals shown to interact directly with DNA, forming Cr-DNA adducts and causing DNA damage. Nickel and cadmium are thought to damage DNA through the inhibition of repair enzymes [5]. Nickel and cadmium deregulate cell proliferation by perturbing various signaling pathways and transcription factors, possibly through reactive oxygen species, although the activation of these pathways is poorly understood [5].

While these metals are known to cause adverse health effects and to be toxic to the lungs, kidneys, liver, and other vital organs [7], [8], the genes and toxicity pathways that respond to metal exposure are not completely known. Therefore, to further elucidate common and unique mechanisms of toxicity and identify the genes involved in the perturbed pathways, we performed side-by-side comparisons of the effects of nickel, cadmium, and chromium in H4-II-E-C3 cells using Affymetrix DNA microarrays. H4-II-E-C3 cells were selected for use as they are well characterized and metabolically active liver models [9]. The cells were exposed to nickel (II) chloride (NiCl_2_), cadmium chloride (CdCl_2_), or sodium dichromate (Na_2_Cr_2_O_7_). We identified 992 probe sets whose expression is affected by exposure to at least one of the metals (430 in nickel, 456 in chromium, and 288 in cadmium). In the comparison study here, we demonstrated that the metals were able to elicit distinct changes in the gene expression profiles, and we identified both common and unique mechanisms of toxicity among the metals.

## Materials and Methods

### Cell Culture Conditions and Exposures

H4-II-E-C3 cells (ATCC, Manassas, VA) were grown in Dulbecco's Modified Eagle's Medium (DMEM; Lonza, Walkersville, MD) containing 10% fetal bovine serum (Invitrogen, Carlsbad, CA) and 10 mL Glutamax (Invitrogen) in T75 flasks incubated at 37°C with 5% carbon dioxide. Exposures were initiated once flasks were 90±10% confluent using the test chemicals NiCl_2_, CdCl_2_, and Na_2_Cr_2_O_7_ (Sigma-Aldrich, St. Louis, MO). Exposure concentrations were chosen based on the CellTiter-Fluor Cell Viability and CellTiter 96 Aqueous Non-Radioactive Cell Proliferation Assays (Promega, Madison, WI) at a no observed cell death level, and at the 20% and 50% cell death levels corresponding to 40, 140, and 400 µM for NiCl_2_; 0.275, 1, and 10 µM for Na_2_Cr_2_O_7_; and 0.2, 0.55, and 1.2 µM for CdCl_2_ (Figure S1). Prior to exposure, flasks were washed twice with serum free DMEM to remove residual serum components with a five minute incubation between washes. Fifteen milliliters of serum free DMEM containing the proper concentration of toxicant were then added to each flask for 24 hours. Serum free medium was used as we are conducting a parallel study examining secreted proteins, and proteins in serum would interfere with this analysis. Four biological replicates were performed for each condition, including an unexposed control.

### RNA Extraction

The cells were scraped from the surface of the flasks and were homogenized using a Dounce homogenizer in Trizol solution (Invitrogen). Total RNA was extracted using Trizol solution per the manufacturer's instructions. An RNeasy Midi Kit cleanup (Qiagen, Germantown, MD) was performed per the manufacturer's instructions to remove residual salts and organic solvents. RNA quality and quantity were determined using the Agilent Bioanalyzer Series II RNA 6000 Nano LabChip Kit and 2100 Bioanalyzer (Agilent, Palo Alto, CA).

### Microarray Preparation and Processing

cDNA and labeled cRNA were prepared using the Affymetrix GeneChip® Two-Cycle Target Labeling kit and 7.5 µg total RNA according to the GeneChip Expression Analysis Technical Manual (701021 Rev. 5). Twenty micrograms of biotin-labeled cRNA was sent to the laboratory of Dr. Maryanne Vahey at the Walter Reed Army Institute of Research Vaccine Genomics Laboratory for processing and scanning on the GeneChip Rat Genome 230 2.0 Array using Affymetrix instrumentation according to the manufacturer's instructions (Affymetrix, Santa Clara, CA).

### Data Analysis

Microarray data was processed for background adjustment, normalization, and summarization using the Robust Multi-Array Averaging method (RMA) [10] using Partek Genomic Suite (GS) software (Version 6.4 Copyright 2009, St. Louis, MO). All data is compliant with the Minimum Information About a Microarray Experiment (MIAME) guidelines and the raw data files can be found in the NCBI Gene Expression Omnibus (accession number GSE31503). The microarray data was examined for outliers using a principal component analysis (PCA) in Partek GS. Pairwise correlation analysis and inter-replicate dot plots of all probe sets were performed to verify reproducibility. Replicates were accepted with an R^2^>0.95 and no gross deviations from linearity. If a sample did not meet these criteria, a new microarray was processed from the total RNA. A present, absent, or marginal detection call for each probe set was determined using the Affymetrix GCOS algorithm, and only probe sets with a “present” detection call for all samples in at least one condition were retained for analysis [11].

An analysis of variance (ANOVA) was performed to determine which genes were differentially expressed due to treatment. The 16,026 probe sets that met the present detection call criteria were analyzed using 2-way ANOVAs (dose and batch) with contrasts for each exposure concentration versus the control using Partek GS for each metal. The batch variable was included to control for differences observed in the PCA resulting from different experimental and processing dates. Probe sets with a Benjamini and Hochberg False Discovery Rate (FDR) [12] less than or equal to 0.001 for the concentration variable and a 1.8 or greater fold change from control in at least one treatment condition were retained for bioinformatic analysis.

After an initial unsupervised ontology analysis, it was observed that multiple related categories were present in the results that were similar to known effects of these metals. Therefore, a manual binning method was devised in order to attribute intuitive biological functions to a large portion of differentially expressed genes. This scheme assigned the major biological processes that were modulated by treatment with the toxicants by developing groups, or “bins”, based on multiple Gene Ontology (GO) categories that correspond to the known effects of the metals. Seven bin categories were created: cell cycle, oxidative stress, ion homeostasis, apoptosis, energy regulation, hypoxic response, and DNA damage, replication, and repair. Each bin was compromised of multiple, related GO terms based on the GO biological process terms provided by Affymetrix in the annotation file (build 29, 2009-7-13) for the Rat Genome 230 2.0 Array. The GO terms found in each bin can be found in Table S1. Probe sets were assigned to a bin if the GO term associated with that probe set was also contained in that particular bin. A chi-squared test was used to test bin enrichment (*p*≤0.05), comparing the differentially expressed probe sets in a bin against all the probe sets called “present” (see Data Analysis above) and having the ontology terms for included in the bin. Probe sets that did not contain any biological process annotation were not considered for significance testing.

Differentially expressed probe sets were clustered using VxInsight and VxArrayImport 0.2.5 with default settings [13] (Sandia National Laboratories, Albuquerque, NM) to identify probe sets with similar expression patterns among all chemical exposures. VxInsight uses a force directed placement algorithm to move similar items closer together while simultaneously pushing dissimilar objects away from each other, and then displays the relationships on a 3D terrain-like map [14]. Clusters were manually selected by their natural boundaries using the terrain view. Transcription factor enrichment for probe sets in each cluster and metal were investigated using MetaCore (GeneGo, St. Joseph, MI).

Ingenuity Pathway Analysis (IPA) software (Ingenuity Systems, www.ingenuity.com, analysis date 2009-11-09) was used to explore the biological implications of the data. Core analyses were performed on the data using the Rat Genome 230 2.0 Array as the reference set with all other default settings selected. We considered canonical pathways, which are well characterized metabolic or cell signaling pathways that are drawn based on the IPA Knowledge Base, statistically significant with a p-value≤0.05.

### qPCR Validation

Quantitative polymerase chain reaction (qPCR) was used to validate a subset of the microarray results from the toxicant exposures (Figure S2). The total RNA from the toxicant exposures used for microarray analysis was also used for qPCR validation. Care was taken to choose genes that were over-expressed or repressed by each of the three chemicals and these genes are listed in Figure S2. The primers were designed using Primer Express software (Applied Biosystems, Foster City, CA) based on the National Center for Biotechnology Information Reference Sequence mRNA. cDNA was prepared from total RNA using the Advantage RT-for-PCR kit per the manufacturer's instructions published April 2006 (Clonetech, Mountain View, CA). The Applied Biosystems SYBR Green Master Mix was used in a 50 µl qPCR reaction with 2 µl of cDNA template and a 2.5 µM final concentration of each primer. A DNA Opticon 2 (Bio-Rad, Hercules, CA) was used for thermal cycling and fluorescence detection using the following scheme: 95°C for 10 minutes followed by 40 cycles of: 95°C for 15 seconds, 60°C for 1 minute, and a fluorescence signal read. Relative fold change was determined using the comparative C_t_ method using beta actin and glyceraldehyde-3-phosphate dehydrogenase as endogenous controls [15]. Values from the four biological replicates were averaged.

The microarray results were compared to the qPCR results using Pearson's product-moment correlation coefficient as well as fold change comparisons (Figure S2). A fold change of 1.5 determined by qPCR in the same direction as the identified differential expression in the microarray data was considered a successful validation. The only gene that did not meet this criteria was the lactate dehydrogenase A gene in the nickel high dose, although the mid dose did meet the fold change criterion.

## Results and Discussion

Since nickel, cadmium, and chromium are potential environmental and occupational hazards, we undertook a study to identify common and unique mechanisms of toxicity for the three metals with a focus at the level of gene expression and molecular pathways. We exposed a rat hepatoma derived cell line (H4-II-E-C3) to three concentrations of NiCl_2_, CdCl_2_, or Na_2_Cr_2_O_7_ for 24 h and then analyzed for transcriptional changes using whole genome DNA oligonucleotide microarrays. Pathways and biological functions affected by the exposure to each metal were indentified and then compared among the metals to further explore similarities and differences in the responses to the three metals.

### Microarray Analysis

**Figure 1:**
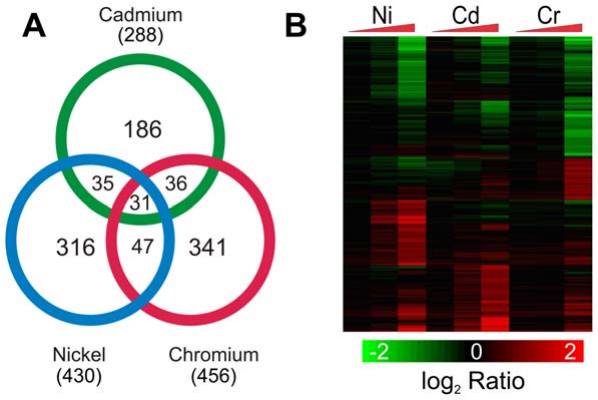
Nickel, chromium, and cadmium exposures affect different groups of genes. (A) An ANOVA was used to identify differentially expressed probe sets using an FDR<0.001 and a 1.8 fold change filter. In total, 992 probe sets were differentially expressed in at least one metal exposure. The differentially expressed genes may be found in Tables S2 and S3. (B) Hierarchical clustering demonstrates that some probe sets respond to only a single metal while others respond to two or three. Dose dependent responses are evident. Each column represents a treatment condition and each row represents an individual probe set. The triangles indicate increasing concentrations of metals and the color indicates the log_2_ ratio of control to experimental expression levels.

To identify genes differentially expressed due to exposure to the metals, we measured mRNA levels using whole-genome, DNA oligonucleotide microarrays. The data was preprocessed using the RMA method and filtered to select only probe sets with a present call in all replicates of at least one condition; 16,311 out of the 31,099 possible probe sets were retained for further analysis. Differentially expressed genes were identified by calculating two-way ANOVAs (dose and batch) for each metal independently. The differentially expressed probe sets were selected using a Benjamini-Hochberg FDR ≤0.001 and a fold change filter of ≥1.8 in at least one treatment condition for each metal, which identified of 430, 456, and 288 probe sets in nickel, chromium, and cadmium, respectively (Table S2 and S3). Many of the probe sets are differentially expressed in more than one chemical exposure, yielding a total of 992 differentially expressed probe sets (Figure 1) taking overlaps between conditions into account.

A concentration dependant response is evidenced by the increase in differential expression as can be observed in the heat map of modulated genes (Figure 1). With the stringent criteria used to identify changes, all of the differentially expressed genes used for analysis are in the highest concentrations of each metal. For all three metals, no genes are differentially expressed at the lowest concentration. In the middle concentration for nickel, 13 probe sets are differentially expressed, all of which are also differentially expressed in the highest concentration. In the middle concentration of chromium, no probe sets are differentially expressed. Only one probe set is differentially expressed in the middle concentration of cadmium and is also identified in the highest concentration.

**Figure 2:**
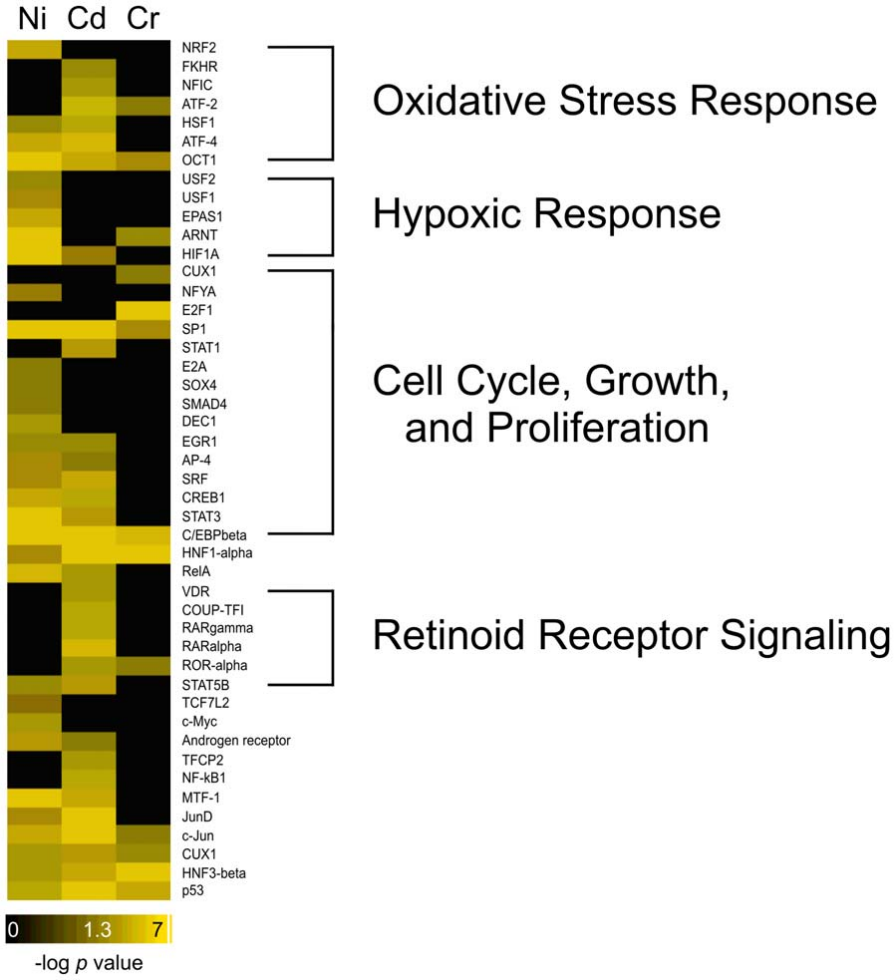
Transcription factor enrichment analysis. Enrichment analysis conducted using MetaCore shows transcription factors whose targets are overrepresented in the differentially expressed gene lists. Transcription factors involved in the oxidative stress response, hypoxic response, cell cycle, cell growth and proliferation, and retinoic acid signaling were enriched. The values are presented as the –log of the p-value.

**Figure 3:**
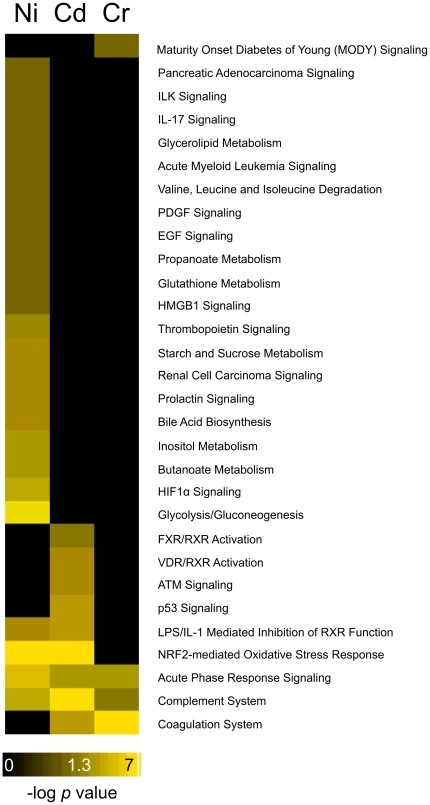
Canonical pathways associated with differentially expressed genes. IPA canonical pathways are enriched by the genes differentially expressed by exposure to nickel, chromium, and cadmium. While overall mechanisms of the three metals are similar, unique and common pathways are identified. The values are presented as the –log of the p-value.

With the goal of comparing the response to these metals at the mechanistic level, a variety of enrichment analyses were performed to identify biological processes that were statistically over-represented in the differentially expressed gene lists. Standard enrichment analyses were performed using MetaCore software to identify transcription factors potentially associated with differentially expressed genes in our data set (Figure 2) and Ingenuity Pathway Analysis software to identify canonical pathways (Figure 3). We discovered a number of enriched transcription factors involved in DNA damage response, cell cycle, cell growth and proliferation, oxidative stress, and hypoxia. The canonical pathways enriched in our data include processes related to the hypoxic response, glutathione metabolism, oxidative stress, and retinoid receptor signaling. Upon initial review of these findings and the differentially expressed gene lists, it was evident that several biological processes are represented, but conventional gene ontology and pathway categories failed to capture the complexity of these responses. In order to provide a more comprehensive view, we created gene ontology bins which include multiple gene ontology terms that are involved in the same biological process and calculated enrichment statistics on these bins (Table 1). The processes that are enriched in our data set include oxidative stress, DNA damage, apoptosis, hypoxic response, and energy regulation. These are consistent with many of the known mechanisms of toxicity for the three metals and provide a starting point to allow us to compare and contrast the response among the metals.

**Table 1:** Enriched ontology bins by metal.

	Total	Nickel		Chromium		Cadmium	
Ontology Bin	Probe Sets	Probe Sets	*p* Value	Probe Sets	*p* Value	Probe Sets	*p* Value
**DNA Damage**	623	10	0.974	44	<0.001	8	0.910
**Oxidative Stress**	490	29	<0.001	29	0.002	19	0.014
**Apoptosis**	635	23	0.447	17	0.701	25	0.004
**Energy**	143	15	<0.001	5	0.953	8	0.008
**Hypoxia**	117	9	0.005	5	0.608	4	0.404

Bins were created to identify the function of a large number of differentially expressed genes and are based on known effects of nickel, chromium and cadmium. Probe sets are assigned to bins based on gene ontology biological process terms. A chi-squared test was used to determine whether the proportion of probe sets in a bin due to metal exposure differed from the proportion of probe sets in each bin based on probe sets having a “present” call (see Methods) in the data set and at least one ontology term from the relevant bin. The oxidative stress bin was enriched in response to all three metals, while a large proportion of probe sets modulated by chromium were assigned to the DNA damage bin, and nickel enriched the hypoxic response and energy regulation bins.

**Figure 4:**
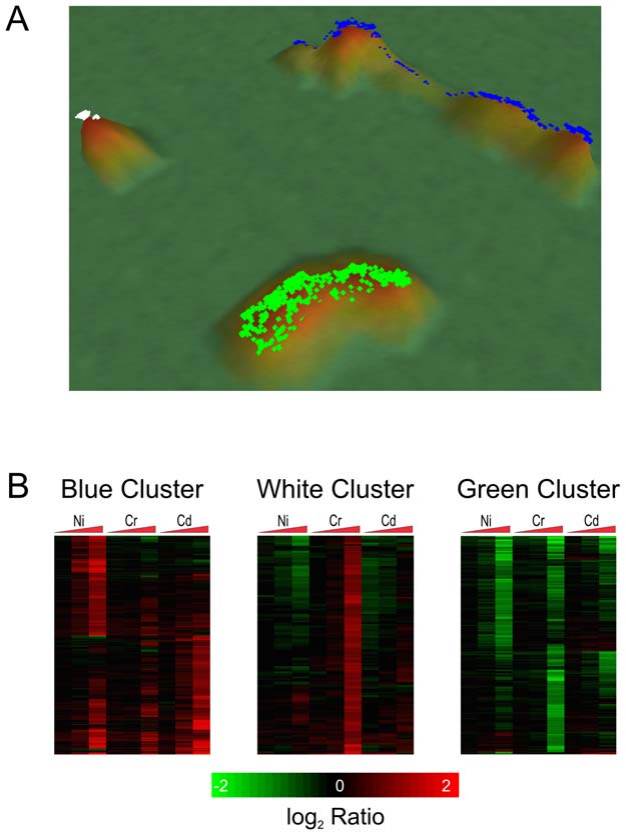
Cluster analysis of 992 differentially expressed probe sets. (A) Cluster analysis of all differentially expressed probe sets was performed using VxInsight, which clusters together probe sets with similar expression patterns, suggesting they may be co-regulated. Each colored point represents one probe set, and the height of each peak is proportional to the number of data points beneath it. Probe sets with similar expression patterns among the metal exposures cluster closely together while those that are different are further apart. Three clusters are indicated by white, blue, and green dots. (B) The expression patterns are depicted by the heat map with the triangles indicating increasing concentrations of metals and the color indicating the log_2_ ratio of control to experimental expression levels. The white cluster is comprised almost entirely of probe sets up-regulated by exposure to chromium.

As an additional step in categorizing the responses to the toxicants, we performed a cluster analysis among differentially expressed genes across all three of the metal exposures to identify potentially co-regulated genes using VxInsight [16]. Three clusters were identified which are highlighted in white, blue, or green (Figure 4), and contain 129, 456, or 407 probe sets respectively. The blue and green clusters are primarily comprised of probe sets that are up- and down-regulated, respectively, but provide no metal specificity. The white cluster is striking since it is tightly clustered, further away from the others, and comprised almost entirely of probe sets up-regulated in response to chromium. Many of the genes in this cluster are involved in the response to DNA damage as discussed below.

### Common Response

**Figure 5:**
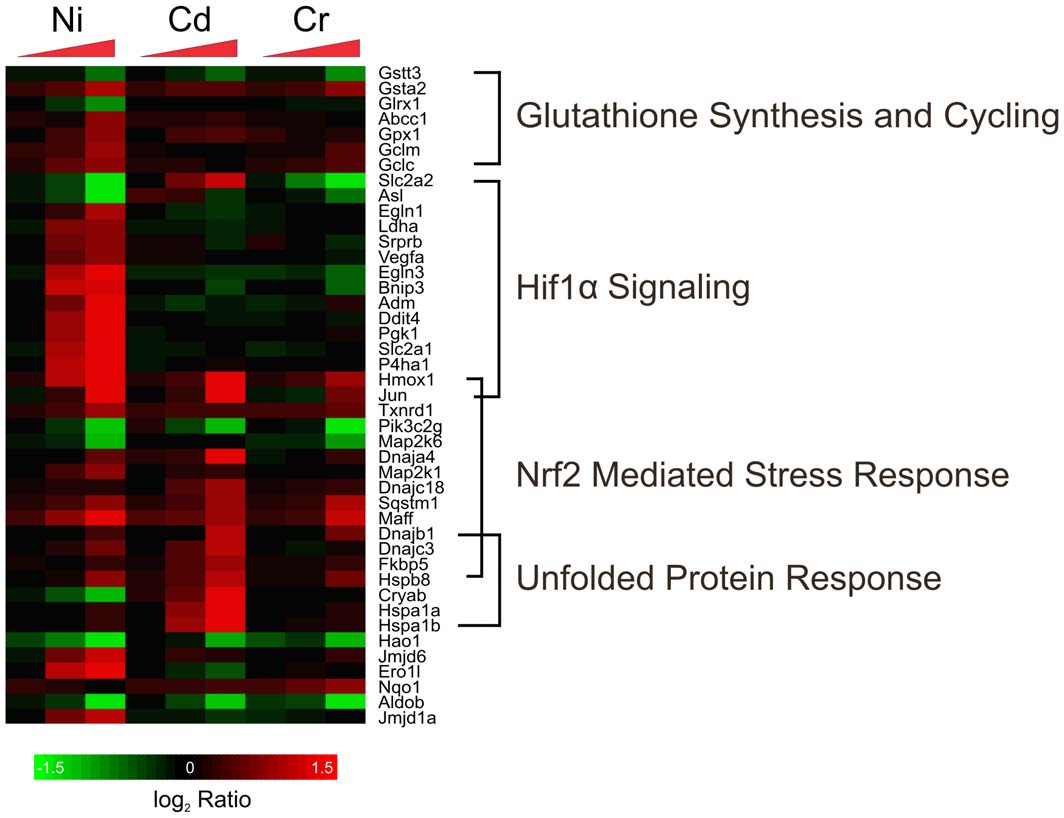
Expression Patterns of genes involved in the response to oxidative stress. The expression patterns of 43 genes that are involved in the oxidative stress response are depicted. While all three metals share the common overall mechanism and show a Nrf2 mediated stress response, nickel-induced genes involved in glutathione synthesis, and cadmium induced those that may be responding to ER stress. The triangles indicate increasing concentrations of metals and the color indicates the log_2_ ratio of control to experimental expression levels.

One particular interest for us was to identify processes that are common to all of the metals. In these experiments, the only perturbed biological process common to all three metals is oxidative stress, a known effect of each of these metals, as evidenced by the oxidative stress bin being enriched in response to the three metals (Table 1) [5]. While some of the changes in gene expression are consistent across the metals, our observations suggest that there are also subtle variations in how the cells respond to what is presumed to be a common mechanism of toxicity. The most notable differences are the modulation of genes involved in the production of the anti-oxidant protein glutathione in response to nickel and ROS-induced endoplasmic reticulum (ER) stress in response to cadmium (Figure 5) [17].

#### Oxidative Stress

The nuclear factor erythroid 2-related factor 2 (Nrf2) oxidative stress response appears to be activated in response to all three metals. The canonical pathway is statistically enriched only for nickel and cadmium, and the Nrf2 transcription factor is significantly enriched only in the nickel data. However, key Nrf2 controlled genes are up-regulated in samples from all three metals, including *Hmox1, Sqstm1,* and glutathione-*S*-transferases. Taking these three separate pieces of evidence together, we conclude that the Nrf2 mediated response is activated in response to all three metals, even if statistical significance is not met in all of the analysis methods. Nrf2 is a transcription factor that controls the expression of important detoxification and oxidative stress proteins [18], [19]. HMOX1 is a ubiquitous stress response protein involved in reducing the effects of oxidative stress and apoptosis [20]. Sequestosome 1 (SQSTM1) has been shown to play a role in the sustained activation of Nrf2 in response to oxidative stress [21].

Other genes and transcription factors known to respond to oxidative stress outside of the Nrf2 pathway are also differentially regulated by all three metals. For example, *Hao1* is down-regulated. The enriched transcription factors include FKHR and NFIC for cadmium, HSF1 and ATF-4 for both nickel and cadmium, and OCT1 for all three chemicals. HAO1 is a liver specific enzyme that converts α-hydroxy acids to α-keto acids while reducing molecular oxygen to H_2_O_2_, and has been shown to be down-regulated due to oxidative stress [22]. FKHR has been shown to be a principal component in the response to oxidative stress by stimulating the expression of metal containing antioxidant proteins [23]. HSF1 decreases intracellular reactive oxygen species generation, thereby protecting against further damage [24].

The enrichment of the Nrf2-mediated oxidative stress response canonical pathway and the modulation of key genes known to respond to oxidative stress suggest that all three metals induce oxidative stress, with chromium showing the lowest level of induction. The metals do, however, differentially affect other mechanisms that control oxidative stress.

Glutathione pathways appear to be activated only in response to nickel. The IPA Glutathione Metabolism canonical pathway (Figure 3) is significantly enriched due only to exposure to nickel, and a number of the genes affected solely by nickel are involved in the protection of the cell by glutathione (Figure 5), including glutamate-cysteine ligase, modifier subunit (*Gclm*) and ATP-binding cassette sub-family C member 1 (*Abcc1*). GCLM is the first and rate limiting enzyme of glutathione synthesis and ABCC1 has been shown to be a glutathione transporter [25], [26]. The role of glutathione in response to nickel toxicity is likely two-fold; both as an antioxidant and in neutralizing the toxic effects of nickel by acting as a chelator, thus increasing the efflux of nickel out of the cell [27]. This up-regulation of glutathione metabolism suggests a mechanism unique to nickel.

Oxidative stress in cadmium exposed cells leads to ER stress including the induction of the unfolded protein response and apoptosis. A number of genes modulated solely by cadmium are indicative of the unfolded protein response, a mechanism not seen in response to nickel or chromium (Figure 5). Six genes encoding chaperones (*Hspa1a*, *Hspa1b*, *Hspb8, Dnajb1, Dnajc3, and Cryab*) are up-regulated. Chaperone proteins are known to be involved in apoptosis as well as the folding and degradation of damaged proteins in the unfolded protein response [28].

ER stress can lead to apoptosis, and our data support the occurrence of apoptosis in cadmium exposed cells. The apoptosis ontology bin is significantly enriched (Table 1), and Caspase 4 (*Casp4)*, encoding an apoptosis-related cysteine peptidase [29], is up-regulated due to exposure to cadmium (Table S3). Two other apoptotic genes, typically seen up-regulated in response to DNA damage, were also up-regulated in the cadmium data (Table S3): a protein phosphatase 1 regulatory (inhibitor) subunit 15A (*Ppp1r15a*), and DNA-damage inducible transcript 3 (*Ddit3*) [30], [31]. However, in this work we believe that the induction of *Ppp1r15a* and *Ddit3* is purely related to their role in apoptosis and not indicative of DNA damage. Overall, these results suggest that cadmium-induced oxidative stress causes ER stress leading to the unfolded protein response and apoptosis.

While the three metals do share some common responses to the disturbance of the cell's normal redox state, each metal affects a unique subset of genes. Chromium appears to have a lower level of induction for the Nrf2 pathway, there is evidence for nickel induced production of the antioxidant glutathione, and cadmium mediates an oxidative stress-induced ER stress characterized by the unfolded protein response and apoptosis.

### Unique Responses

In addition to shared responses, we were interested in identifying mechanisms of toxicity that are unique to each metal. Based on the gene expression changes present in our data, chromium is unique in causing DNA damage; nickel causes a hypoxic response and perhaps disruption of chromatin structure; and cadmium causes a disruption of retinoic acid signaling pathways.

#### Cr-induced DNA Damage

**Figure 6:**
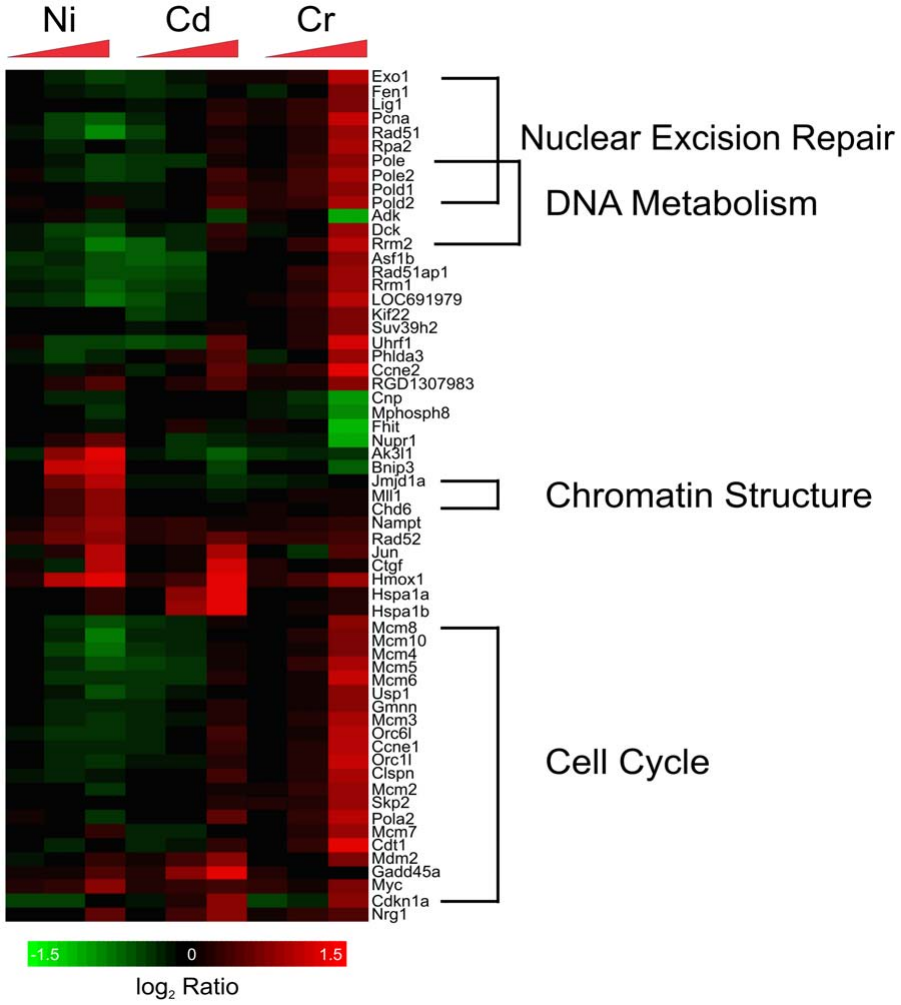
Expression patterns of genes involved in the response to DNA damage and chromatin structure. The expression patterns of 61 genes involved in chromatin structure or the DNA damage response are depicted. Chromium elicited the most extensive response perturbing genes involved in nuclear excision repair, DNA metabolism, and cell cycle. A number of genes involved in chromatin structure modifications were up-regulated only by nickel. The triangles indicate increasing concentrations of metals and the color indicates the log_2_ ratio of control to experimental expression levels.

Chromium is the only metal of the three that clearly appears to cause DNA damage. Our data supports this mechanism with genes involved in DNA repair and replication being modulated by exposure to the chromium (Figure 6) and the DNA damage ontology bin being enriched (Table 1). The transcription factor E2F1, which is induced by DNA damage, plays an important role in DNA repair at stalled replication forks [32]. Strikingly, among the probe sets in the white VxInsight cluster (Figure 4), most of which are induced by chromium, 40 of the 79 probe sets with annotation provided by Affymetrix are involved in the DNA damage response. Many of these genes have products that can be found in the DNA synthesome, which is a multiprotein complex involved in DNA replication [33], [34]. Proliferation cell nuclear antigen (*Pcna*), replication protein A (*Rpa2*), the minichromosome maintenance complex component genes which encode helicases, DNA ligase (*Lig1*), DNA polymerase ε (*Pole and Pole2*), and DNA polymerase δ (*Pold1 and Pold2*) are all up-regulated as a result of exposure to chromium (Figure 6), suggesting that there is an increase in DNA synthesis, likely due to chromium-induced DNA damage.

The proteins that make up part of the synthesome play specific roles in DNA synthesis and repair that are consistent with the known mechanism of chromium-induced DNA damage. Chromium has been shown to directly interact with DNA and cause damage by forming DNA adducts and causing DNA strand breaks [5], [35]. DNA polymerases δ and ε are associated with proof-reading and repair activity [36]. These polymerases, as well as several other proteins including RPA2 and PCNA, may be involved in excision repair to remove DNA adducts. DNA ligase is involved in repairing double strand breaks, which are known to accumulate due to chromium toxicity. Some of the encoded proteins of the genes differentially expressed in the DNA synthesome, such as those forming the minichromosome maintenance complex (MCM) and origin recognition complex (ORC), are involved specifically in the initiation of DNA synthesis [37]. Since it has been shown that chromium-DNA adducts reduce the ability of synthesome to initiate replication [38], the expression of these genes may be up-regulated as the cell attempts to repair damage caused by the chromium. These responses suggest that the direct interaction of chromium with DNA and the formation of adducts and stand breaks are involved in chromium toxicity.

Although nickel, cadmium, and chromium are believed to be at least weakly genotoxic, we did not detect changes in gene expression clearly attributable to DNA-damage in cadmium and nickel exposed cells despite having clear evidence for ongoing DNA replication and repair caused by chromium. The p53 transcription factor is enriched for all three chemicals and the p53 and ATM signaling canonical pathways are enriched due to cadmium exposure. However, the differentially expressed genes involved in these enrichments and the p53 response itself, are not specific to DNA damage and repair. Additionally, the DNA damage and repair specific genes are unique to chromium. Therefore, at the concentrations tested, chromium is the only of the three metals to cause high levels of DNA damage in H4-II-E-C3 cells.

#### Hypoxia and Disruption of Protein Function by Ni

Gene expression changes seen in the nickel-exposed samples were consistent with a hypoxic response. The hypoxic response ontology bin (Table 1), HIF-1α canonical pathway (Figure 3), and HIF-1α transcription factor (Figure 2) are enriched in the nickel data. HIF-1α is a transcription factor which induces the transcription of genes involved in glycolysis, glucose transport, apoptosis, and other cellular process as a result of a change in the intracellular oxygen concentration [5]. Additionally, the glycolysis/gluconeogeneis canonical pathway and energy regulation ontology bins were both enriched (Table 1). These could also be potentially a result of HIF-1α regulation, as hypoxic conditions and HIF-1α activation are known to interfere with cellular energy metabolism such as glycolysis, causing a cell to shift toward nonoxidative forms of ATP production and enhancing production of glycolytic enzymes and glucose transporters [39]. Furthermore, the genes encoding lactate dehydrogenase A (*Ldha*), pyruvate dehydrogenase (*Pdk1*), phosphoglycerate kinase 1 (*Pgk1*) and solute carrier family 2 (facilitated glucose transporter) member 1 (*Slc2a1*) are up-regulated; all of which contribute to low oxygen energy utilization (Figure 5 and Table S3) [39]. These data suggests that nickel alters the expression of known HIF-1α targets and induces a hypoxic-like response. The cause of this hypoxic-like response in the case of nickel exposure may not be low oxygen levels. It is thought that nickel activates HIF-1α by preventing the degradation of the protein either through the depletion of ascorbate or by replacing iron in the hydroxylases responsible for HIF-1α degradation [40], [41].

A number of the genes up-regulated specifically by nickel are involved in chromatin structure modifications (Figure 6), including two jumonji family histone demethylases (*Jmjd1a* and *Jmjd6*). It has been shown that nickel can inactivate jumonji family histone demethylases by replacing iron in the enzyme's active site, and the increase seen may be due to this inactivation and not DNA damage [42]. This is a second example of nickel disrupting the normal function of a protein.

Chromatin structure and the hypoxic response are affected by nickel exposure alone. While these two functions are clearly unrelated, they are both mediated by enzymes that require iron as a cofactor. It is known that nickel can substitute for iron in many enzymes and block their function [43]. In nickel exposed cells, Ni/Fe substitution may instigate the induction of the hypoxic response and changes in expression of genes related to chromatin structure. Nickel's ability to disrupt the normal function of these proteins is a major contributor to nickel toxicity that is unique amongst the metals in this study.

#### Retinoic acid signaling

The data also suggests a mechanism unique to cadmium: retinoic acid signaling. The IPA canonical pathways FXR/RXR Activation and VDR/RXR Activation were significant only in response to cadmium (Figure 3). Transcription factors comprised of the retinoid family receptors including RAR gamma, RAR alpha, ROR alpha, and the vitamin D receptor (VDR) are enriched in response to only cadmium (Figure 2). Retinoic acid is a hormone-like molecule that is involved in the regulation of cell differentiation and proliferation whose effects are mediated by retinoic acid receptors [44]. It has been suggested that cadmium acts as an environmental teratogen by increasing the amount of retinoic acid through interference with the retinoic acid metabolizing genes [45]. These enriched pathways and transcription factors suggest that the disruption of retinoid family signaling is a cadmium specific mechanism.

### Unexpected Findings

While we were able to identify both common and unique responses to nickel, cadmium, and chromium, each metal also has known mechanisms of toxicity that we expected our data to reflect based on the literature, but were not apparent. We did not find evidence of nickel- and cadmium-induced DNA damage or a strong induction of oxidative stress by chromium, all of which are well documented effects in other systems.

Nickel, cadmium, and chromium have all been shown to be mutagenic; in our data, however, only chromium appeared to be genotoxic. The DNA ontology bin (Table 1) and E2F1 transcription factor (Figure 2) are significant only for chromium, and genes involved in DNA metabolism were up-regulated only in response to chromium (Figure 6). Chromium can directly interact with and damage DNA, while nickel and cadmium only indirectly damage DNA through the formation of reactive oxygen species and by interfering with DNA repair enzymes. Our inability to detect evidence of nickel and cadmium-induced DNA damage may be a result of these mechanisms; a 24 hour exposure period may not have been long enough for DNA damage to accumulate in H4-II-E-C3 cells.

We also did not observe evidence of a strong induction of oxidative stress due to exposure to chromium. While the Nrf2 oxidative stress response is enriched in the cadmium and nickel data (Table 1; Figure 5), and the Nrf2 transcription factor is enriched in response to nickel (Figure 2), neither are significantly enriched in response to chromium. Also, the change in magnitude of some of the key genes involved in the response to oxidative stress is not as large for chromium as it is for nickel and/or cadmium. For example, *Hmox* is increased almost 7 and 4.5 fold in response to nickel and cadmium (high dose), respectively, but only 2 fold due to chromium (high dose) (Figure 5). The lack of evidence supporting oxidative stress due to chromium as compared to nickel and cadmium is surprising as the formation of reactive oxygen species as Cr(VI) is reduced to Cr(III) intracellularly is well documented [46]. Perhaps at the concentrations used in this work, chromium-induced DNA damage was the dominant effect of the metal, thus overshadowing the oxidative stress response, or the response may be unique to H4-II-E-C3 cells.

### Caveats of analysis

In evaluating the results of our analysis, there are several important caveats worth noting. The first is that the number of enriched categories appears somewhat smaller in the chromium data set, which we believe might be due in part to a weakness in the enrichment analysis approach. The second is that the high overlap in genes across many pathways may lead to the statistical enrichment of processes which are truly uninvolved. The final is the need for equipotent concentrations across the metals to allow a realistic comparison of the toxic mechanisms.

In the IPA canonical pathway analysis for chromium, only 4 pathways were significant compared to 9 and 24 from cadmium and nickel, respectively, and we observed a paucity of enriched transcription factors due to chromium exposure as compared to the nickel and cadmium exposures. Since a large proportion of the chromium modulated genes are involved in the DNA damage response, it may have dominated the enrichment analysis, masking other biologically important perturbed processes. If the DNA damage genes are removed from the chromium analysis, additional canonical pathways achieve statistical significance, including Glycerolipid Metabolism, Glycolysis, Starch and Sucrose Metabolism, and FXR/RXR Metabolism (data not shown). Moreover, applying strict criteria for differential expression as was done in this work can restrict the number of enriched pathways. Less stringent criteria for differential expression might have increased the number of enriched pathways by increasing the number of genes contributing to the enrichment analysis. The stricter criteria for differential expression used here may have led to a high false negative rate, but our findings are well supported.

The large overlap existing among the genes in different pathways and among the transcription factor target lists can also complicate the interpretation of enrichment analyses. The jun proto-oncogene (*Jun*), considered a “hub” molecule, is present in 85 IPA canonical pathways, and *Hmox* is present in 12. In the transcription factor enrichment analysis factors with similar binding sites, such as USF1 and USF2, and the retinoic acid receptors RAR-gamma, RAR-alpha, and ROR-alpha, are all enriched. It is possible that several of these transcription factors may have been assigned to an individual gene because of a single binding site. A few differentially expressed genes could therefore cause significant enrichment of many different pathways or transcription factors, leading to an incorrect analysis. Thus, information gained from simple enrichment analysis tools must be viewed with caution. To prevent the inclusion of spurious processes, we have analyzed individual genes within the enriched categories to help ensure that the results reported here are biologically relevant.

An extremely important, yet challenging, component of performing a comparative toxicogenomic analysis is setting equipotent stimuli across the study conditions. Viability assays, such as those used in our range finding, do not necessarily correspond with or have similar sensitivity as the measurement of gene transcripts. Additionally, each metal affects the cells differently, and concentrations of the metals at equal levels of cytotoxicity may not have the same effect at the gene expression or biological process level. The same biological processes could be perturbed at different levels of cytotoxicity for the different metals. At the concentrations we tested, similar numbers of genes were differentially expressed among the metal exposures. Therefore, based on the similar number of differentially expressed genes and the similar levels of cytotoxicity, we believe that we approximated equipotency sufficiently well to produce useful results.

### Conclusion

Nickel, chromium, and cadmium are heavy metals commonly found in industry use and in the environment which have adverse health effects. In order to identify common and unique molecular mechanisms of toxicity for each metal, a microarray study was performed using rat hepatoma-derived cells exposed to the metals. Nickel, cadmium, and chromium all induced common effects when broadly viewed, but the detailed mechanisms and pathways involved were unique to the metals. All three metals cause oxidative stress, and the cells response to it was mediated at least in part through the Nrf2 transcription factor. However, the oxidative stress response was distinct for each metal. Chromium had the lowest level of response, nickel induced synthesis of the anti-oxidant glutathione, and cadmium led to ROS mediated ER stress and the unfolded protein response. Further, all three metals are known to be genotoxic, yet in this work, only chromium caused extensive stimulation of DNA repair mechanisms, likely through DNA adduct formation and DNA strand breakage. Nickel induced disruption of the normal function of proteins causing Hif-1α activation and disruption of chromatin structural proteins was a mechanism unique to this metal. Cadmium caused disruption of retinoic acid signaling, which is a likely mechanism for cadmium-induced teratogenicity.

In conclusion, the gene expression of the H4-II-E-C3 cell line was investigated to identify mechanisms of toxicity for nickel, chromium, and cadmium. Identified mechanisms included oxidative stress, DNA damage, disruption of protein function, and disruption of retinoic acid signaling. While microarray analysis suggests toxicity pathways involved in heavy metal intoxication, further investigation will be required to verify these findings. This work provides a starting point for future studies by providing key genes and transcription factors that may be directing the cells' response to toxic insults by nickel, chromium, or cadmium.

## Supporting Information

Figure S1 **Cell viability assay results for rangefinding.** Rangefinding studies were conducted to calibrate the metal concentrations in the definitive exposures. Exposure concentrations were chosen at the 0, 20, and 50 percent effect level of each metal based on a cell viability assay.
(XLS)

Figure S2 **Comparison of fold changes determined by qPCR and microarray.** A subset of differentially expressed genes was validated through qPCR. Fold changes were compared and a Pearson product-moment correlation coefficient was calculated between the qPCR and array fold changes.
(XLS)

Table S1 **GO Terms contained in each bin.** Bins, based on multiple Gene Ontology (GO) categories provided in the Affymetrix annotation file that correspond to the known effects of the metals, were created to describe function to a large number of probe sets. Seven bins were created: cell cycle, oxidative stress, ion homeostasis, apoptosis, energy regulation, hypoxic response, and DNA damage, replication, and repair. The GO terms contained in each bin are listed.
(XLS)

Table S2 **Differentially expressed probe sets by metal.** Probe sets with a *p*<0.001 and are changing by at least 1.8 fold are listed in each worksheet labeled for the metal in which they are differentially expressed, representing a total of 992 probe sets. The VxInsight cluster for each probe set is also listed.
(XLS)

Table S3 **Differentially expressed gene with log_2_ ratio of change for each condition.** Probe sets with a *p*<0.001 and are changing by at least 1.8 fold are listed representing a total of 992 probe sets. The average log_2_ ratio of change from the unexposed samples is listed for each exposure condition.
(XLS)

## References

[1] Agency for Toxic Substances and Disease Registry (2005). Toxicological profile for nickel.

[2] Cohen MD, Kargacin B, Klein CB, Costa M (1993). Mechanisms of chromium carcinogenicity and toxicity.. Crit Rev Toxicol.

[3] Salnikow K, Zhitkovich A (2008). Genetic and epigenetic mechanisms in metal carcinogenesis and cocarcinogenesis: nickel, arsenic, and chromium.. Chem Res Toxicol.

[4] Jarup L, Akesson A (2009). Current status of cadmium as an environmental health problem.. Toxicol Appl Pharmacol.

[5] Beyersmann D, Hartwig A (2008). Carcinogenic metal compounds: recent insight into molecular and cellular mechanisms.. Arch Toxicol.

[6] Stohs SJ, Bagchi D (2008). Oxidative mechanisms in the toxicity of metal ions.. Free Radical Biology & Medicine.

[7] Jarup L, Berglund M, Elinder CG, Nordberg G, Vahter M (1998). Health effects of cadmium exposure--a review of the literature and a risk estimate.. Scand J Work Environ Health.

[8] Sunderman FW, Marzouk A, Hopfer SM, Zaharia O, Reid MC (1985). Increased lipid peroxidation in tissues of nickel chloride-treated rats.. Ann Clin Lab Sci.

[9] Michels G, Watjen W, Weber N, Niering P, Chovolou Y (2006). Resveratrol induces apoptotic cell death in rat H4IIE hepatoma cells but necrosis in C6 glioma cells.. Toxicology.

[10] Irizarry R, Hobbs B, Collin F, Beazer-Barclay Y, Antonellis KJ (2003). Exploration, normalization, and summaries of high density oligonucleotide array probe level data.. Biostatistics.

[11] Archer KJ, Reese SE (2010). Detection call algorithms for high-throughput gene expression microarray data.. Brief Bioinform.

[12] Benjamini Y, Hochberg Y (1995). Controlling the false discovery rate: a practical and powerful approach to multiple testing.. Journal of the Royal Statistical Society.

[13] Martinez MJ, Roy S, Archuletta AB, Wentzell PD, Anna-Arriola SS (2004). Genomic analysis of stationary-phase and exit in Saccharomyces cerevisiae: gene expression and identification of novel essential genes.. Mol Biol Cell.

[14] Boyack KW, Wylie BN, Davidson GS (2002). Domain Visualization Using VxInsight for Science and Technology Management.. Journal of the American Society for Information Science and Technology.

[15] Livak KJ, Schmittgen TD (2001). Analysis of relative gene expression data using real-time quantitative PCR and the 2(-Delta Delta C(T)) Method.. Methods.

[16] Kim SK, Lund J, Kiraly M, Duke K, Jiang M (2001). A gene expression map for Caenorhabditis elegans.. Science.

[17] Hiramatsu N, Kasai A, Du S, Takeda M, Hayakawa K (2007). Rapid, transient induction of ER stress in the liver and kidney after acute exposure to heavy metal: evidence from transgenic sensor mice.. FEBS Lett.

[18] Li W, Kong AN (2009). Molecular mechanisms of Nrf2-mediated antioxidant response.. Mol Carcinog.

[19] Klaassen CD, Reisman SA (2010). Nrf2 the rescue: effects of the antioxidative/electrophilic response on the liver.. Toxicol Appl Pharmacol.

[20] Ryter SW, Choi AM (2009). Heme oxygenase-1/carbon monoxide: from metabolism to molecular therapy.. Am J Respir Cell Mol Biol.

[21] Jain A, Lamark T, Sjottem E, Larsen KB, Awuh JA (2010). p62/SQSTM1 is a target gene for transcription factor NRF2 and creates a positive feedback loop by inducing antioxidant response element-driven gene transcription.. J Biol Chem.

[22] Recalcati S, Tacchini L, Alberghini A, Conte D, Cairo G (2003). Oxidative stress-mediated down-regulation of rat hydroxyacid oxidase 1, a liver-specific peroxisomal enzyme.. Hepatology.

[23] Sidhu A, Miller PJ, Hollenbach AD (2011). FOXO1 stimulates ceruloplasmin promoter activity in human hepatoma cells treated with IL-6.. Biochem Biophys Res Commun.

[24] Zhang L, Jiang H, Gao X, Zou Y, Liu M (2011). Heat shock transcription factor-1 inhibits H_2_O_2_-induced apoptosis via down-regulation of reactive oxygen species in cardiac myocytes.. Mol Cell Biochem.

[25] Rothnie A, Conseil G, Lau AY, Deeley RG, Cole SP (2008). Mechanistic differences between GSH transport by multidrug resistance protein 1 (MRP1/ABCC1) and GSH modulation of MRP1-mediated transport.. Mol Pharmacol.

[26] Franklin CC, Backos DS, Mohar I, White CC, Forman HJ (2009). Structure, function, and post-translational regulation of the catalytic and modifier subunits of glutamate cysteine ligase.. Mol Aspects Med.

[27] Joshi S, Husain MM, Chandra R, Hasan SK, Srivastava RC (2005). Hydroxyl radical formation resulting from the interaction of nickel complexes of L-histidine, glutathione or L-cysteine and hydrogen peroxide.. Hum Exp Toxicol.

[28] Bertin G, Averbeck D (2006). Cadmium: cellular effects, modifications of biomolecules, modulation of DNA repair and genotoxic consequences (a review).. Biochimie.

[29] Binet F, Chiasson S, Girard D (2010). Evidence that endoplasmic reticulum (ER) stress and caspase-4 activation occur in human neutrophils.. Biochem Biophys Res Commun.

[30] Garrido JL, Maruo S, Takada K, Rosendorff A (2009). EBNA3C interacts with Gadd34 and counteracts the unfolded protein response.. Virol J.

[31] Copanaki E, Schurmann T, Eckert A, Leuner K, Muller WE (2007). The amyloid precursor protein potentiates CHOP induction and cell death in response to ER Ca2^+^ depletion.. Biochim Biophys Acta.

[32] Liu K, Lin FT, Ruppert JM, Lin WC (2003). Regulation of E2F1 by BRCT domain-containing protein TopBP1.. Mol Cell Biol.

[33] Jiang HY, Hickey RJ, Abdel-Aziz W, Tom TD, Wills PW (2002). Human cell DNA replication is mediated by a discrete multiprotein complex.. J Cell Biochem.

[34] Coll JM, Sekowski JW, Hickey RJ, Schnaper L, Yue W (1996). The human breast cell DNA synthesome: its purification from tumor tissue and cell culture.. Oncol Res.

[35] Zhitkovich A (2005). Importance of chromium-DNA adducts in mutagenicity and toxicity of chromium(VI).. Chem Res Toxicol.

[36] Tran HT, Gordenin DA, Resnick MA (1999). The 3′-->5′ exonucleases of DNA polymerases delta and epsilon and the 5′-->3′ exonuclease Exo1 have major roles in postreplication mutation avoidance in Saccharomyces cerevisiae.. Mol Cell Biol.

[37] Kawakami H, Katayama T (2010). DnaA, ORC, and Cdc6: similarity beyond the domains of life and diversity.. Biochem Cell Biol.

[38] Dai H, Liu J, Malkas LH, Catalano J, Alagharu S (2009). Chromium reduces the in vitro activity and fidelity of DNA replication mediated by the human cell DNA synthesome.. Toxicol Appl Pharmacol.

[39] Gordan JD, Thompson CB, Simon MC (2007). HIF and c-Myc: sibling rivals for control of cancer cell metabolism and proliferation.. Cancer Cell.

[40] Salnikow K, Donald SP, Bruick RK, Zhitkovich A, Phang JM (2004). Depletion of intracellular ascorbate by the carcinogenic metals nickel and cobalt results in the induction of hypoxic stress.. J Biol Chem.

[41] Maxwell P, Salnikow K (2004). HIF-1: an oxygen and metal responsive transcription factor.. Cancer Biol Ther.

[42] Chen H, Giri NC, Zhang R, Yamane K, Zhang Y (2010). Nickel ions inhibit histone demethylase JMJD1A and DNA repair enzyme ABH2 by replacing the ferrous iron in the catalytic centers.. J Biol Chem.

[43] Kaczmarek M, Timofeeva OA, Karaczyn A, Malyguine A, Kasprzak KS (2007). The role of ascorbate in the modulation of HIF-1alpha protein and HIF-dependent transcription by chromium(VI) and nickel(II).. Free Radic Biol Med.

[44] Mostbock S, Fickova M, Macejova D, Baranova M, Kotyzova D (2002). Effect of cadmium and mercury on the nuclear retinoic acid receptors.. Gen Physiol Biophys.

[45] Cui Y, Freedman JH (2009). Cadmium induces retinoic acid signaling by regulating retinoic acid metabolic gene expression.. J Biol Chem.

[46] Shi X, Chiu A, Chen CT, Halliwell B, Castranova V (1999). Reduction of chromium(VI) and its relationship to carcinogenesis.. Journal of Toxicology and Environmental Health Part B, Critical Reviews.

